# Approaches to Authenticating Products Containing Red Yeast Rice Extract (Monacolin K)

**DOI:** 10.3390/molecules31040723

**Published:** 2026-02-19

**Authors:** Stanislava Ivanova, Velislava Todorova, Daniela Grekova-Kafalova, Zoya Dzhakova, Katerina Slavcheva

**Affiliations:** 1 Department of Pharmacognosy and Pharmaceutical Chemistry, Faculty of Pharmacy, Medical University of Plovdiv, 4002 Plovdiv, Bulgaria; velislava.todorova@mu-plovdiv.bg (V.T.); zoya.dzhakova@mu-plovdiv.bg (Z.D.); 2 Research Institute, Medical University of Plovdiv, 4002 Plovdiv, Bulgaria; 3 PERIMED-2, BG16RFPR002-1.014-0007, Central District, Vasil Aprilov Blvd. 15A, 4002 Plovdiv, Bulgaria; 4 Department of Organisation and Economics of Pharmacy, Faculty of Pharmacy, Medical University of Plovdiv, 4002 Plovdiv, Bulgaria; daniela.kafalova@mu-plovdiv.bg (D.G.-K.); katerina.slavcheva@mu-plovdiv.bg (K.S.)

**Keywords:** cardiovascular disease, food supplements, monacolin K, *Monascus purpureus*, lovastatin, statins

## Abstract

Red yeast rice (RYR) food supplements are widely used for cholesterol management owing to their content of monacolin K (MK), which, in its lactone form, is chemically identical to the prescription statin lovastatin. Despite their popularity, RYR products raise significant quality and safety concerns related to pronounced variability in MK content, frequent labeling non-compliance, contaminations with undeclared pharmaceutical statins, etc. The analytical differentiation between naturally produced MK and added synthetic lovastatin remains particularly challenging due to their identical chemical structures. This review provides a comprehensive overview of the chemical composition of RYR, with emphasis on monacolins, pigments, and relevant secondary metabolites, and critically summarizes current regulatory, safety, and quality issues associated with RYR-based food supplements. Furthermore, a practical, multi-level analytical strategy for product authentication is proposed. The approach integrates targeted quantification of MK and accompanying monacolins, identification of characteristic *Monascus* pigments as authenticity markers, gas chromatography–mass spectrometry for the detection of undeclared statins and other non-declared constituents, and proton nuclear magnetic resonance for global compositional fingerprinting. By combining complementary targeted and non-targeted techniques, this workflow enables more reliable authentication, detection of adulteration, and comprehensive quality assessment. The implementation of standardized analytical protocols is essential to improve transparency and enhance consumer safety in the rapidly expanding RYR supplement market.

## 1. Introduction

Cardiovascular disease (CVD) remains a leading cause of mortality and disability worldwide, representing a major public health challenge [1]. Among modifiable risk factors, dyslipidemia plays a crucial role in atherogenesis and the progression of CVD [1,2]. Although statin therapy has markedly improved cardiovascular outcomes over the past decades, a substantial number of patients remain unable to attain guideline-directed lipid targets and continue to face residual cardiovascular risk [1]. Moreover, certain patient populations have contraindications to statin therapy or are required to discontinue treatment due to serious adverse effects [3,4].

Nowadays, an alternative approach for statin-intolerant patients includes red yeast rice supplementation alongside lifestyle modifications [5]. Red yeast rice (RYR), prepared by fermenting rice with *Monascus purpureus* (*M. purpureus*), a red yeast species, has been utilized for centuries in traditional Chinese medicine [6]. During fermentation, a variety of metabolites are formed, including the pigments responsible for the characteristic red coloration of RYR. This process also leads to the production of monacolins, among which monacolin K (MK), structurally similar to lovastatin, exhibits cholesterol-lowering properties and is considered the principal bioactive compound of RYR [7,8]. Moreover, the cholesterol-lowering use of RYR refers not to traditional dietary red yeast rice, but to food supplements (FSs) standardized for MK content.

FSscontaining MK have gained significant popularity in the past 10 years. The worldwide market for RYR is expected to reach approximately 487.5 million USD in 2025 and is projected to grow to about 862.9 million USD by 2032, reflecting a compound annual growth rate of 8.5% over the 2025–2032 period [9].

Recently, the safety of FSs containing RYR become a highly discussed scientific topic, because of many cases of serious adverse effects following the intake of such products and considerable variability in the composition and quality have been reported [10,11,12,13,14]. Many studies have reported that nearly 20% of these products contain undeclared pharmacologically active ingredients, many of which may expose consumers to serious adverse effects [15,16]. Reports of serious adverse effects, including rhabdomyolysis, hepatitis, and dermatological reactions, have been documented even at daily intakes as low as 3 mg of monacolins from RYR [13].

In the EU and other parts of the World products containing MK are classified as FSs and can be obtained without a prescription [17]. The European Food Safety Authority (EFSA) has indicated that consumption of monacolins from RYR through FSs may result in exposure to MK at doses comparable to therapeutic levels of lovastatin, and that the adverse-effect profile of RYR closely mirrors that of lovastatin [18].

As a result, the use of monacolins from RYR at doses of 3 mg/day or higher was restricted, and the substance was included in Part B (“Restricted substances”) and Part C (“Substances under Community scrutiny”) of Annex III to Regulation (EC) No 1925/2006 [18]. EFSA introduced these regulatory measures after determining that a safe daily intake of monacolins from RYR could not be established without raising concerns for human health, both in the general population and in vulnerable subgroups. Owing to the limited availability of scientific data, substantial uncertainty regarding the safety profile of monacolins from RYR persists. EFSA has therefore invited stakeholders to submit additional evidence demonstrating the safety of these substances in accordance with the applicable EU legislation. On the basis of the data subsequently generated and assessed, the European Commission may decide whether to authorize the use of monacolins from RYR or to include them in Annex III, Part A (“Prohibited substances”) or Part B (“Restricted substances”), of Regulation (EC) No 1925/2006 [18]. The EFSA Panel on Nutrition, Novel Foods and Food Allergens has released updated safety-related information concerning FSs containing RYR. The update confirms that nutrivigilance data on adverse events associated with the consumption of these supplements have been reported following the entry into force of Regulation (EU) 2022/860. This regulation establishes a maximum daily intake of less than 3 mg of monacolins from RYR and restricts their use in certain population groups. Furthermore, a study by Banach et al. (2024) provides data on reported adverse events associated with the consumption of RYR FSs at an intake of 3 mg of MK per day and supports previous safety considerations at this intake level [19,20].

In 2011, EFSA published a scientific opinion establishing a cause-and-effect relationship between the consumption of MK from RYR and the maintenance of normal blood LDL-cholesterol concentrations at a daily intake of 10 mg of MK. On the basis of this conclusion, a health claim relating to MK from RYR and its contribution to the maintenance of normal blood cholesterol levels was authorized and included in the list of permitted health claims laid down in Regulation (EU) No 432/2012. However, in light of the current restriction limiting the daily intake of monacolins from RYR to less than 3 mg, and taking into account the available scientific evidence indicating potential adverse health effects, the European Commission, by means of Regulation (EU) 2024/2041 amending Regulation (EU) No 432/2012, withdrew the authorized health claim for MK from the EU list of permitted health claims [21].

Unlike medicinal products, FSs are generally not subject to mandatory quality testing prior to market release, and regulatory frameworks governing these products are relatively permissive worldwide. In Canada, FSs are classified as natural health products and fall under a specific regulatory framework within the Natural Health Products Regulations, where both products and manufacturers must be licensed, and evidence of safety and efficacy is required. In contrast, in Europe and the United States, there are no pre-market safety requirements, and responsibility for product safety lies primarily with the manufacturer. Within the European Union (EU), FSs are regulated as foods under Directive 2002/46/EC, with legislation largely focused on vitamins and minerals. Regulatory oversight mainly addresses general product integrity, leaving a high risk of adulteration. Furthermore, there are no mandatory requirements for testing the quality or quantity of active compounds, nor for the assessment of impurities, related substances, or heavy metals such as lead [16]. However, patients often assume that these products are safe due to their widespread availability and non-prescription status [22,23].

RYR is considered a product with a high risk of contamination by the nephrotoxic mycotoxin citrinin, which may also be produced by *M. purpureus* [24]. In addition, some RYR supplements have been reported to contain undeclared simvastatin, and RYR is increasingly incorporated into multi-ingredient botanical preparations whose combined effects are not fully evaluated [24,25].

A critical analytical challenge in the quality control of RYR-based FS is the inability of conventional chemical analysis (high-performance liquid chromatography coupled with mass spectrometry (HPLC/MS), nuclear magnetic resonance (NMR), etc.) to reliably distinguish between naturally occurring MK and synthetic lovastatin, due to their identical chemical structures. This analytical limitation creates a significant predisposition for the marketing of counterfeit or adulterated FS, in which pharmaceutical-grade lovastatin may be deliberately added and falsely presented as naturally derived MK. In the absence of an advanced and complex analytical strategy, this issue represents a major obstacle to effective regulatory oversight and poses a substantial risk to consumer safety.

The aim of this article is to present a comprehensive and simple multi-level analytical approach for the authentication of RYR products.

## 2. Chemical Composition of RYR

RYR contains a diverse array of chemical compounds, including monacolins, pigments, organic acids, amino acids, sterols, decalin derivatives, flavonoids, lignans, coumarins, terpenoids, polysaccharides and others [6].

### 2.1. Monacolins

**Figure 1:**
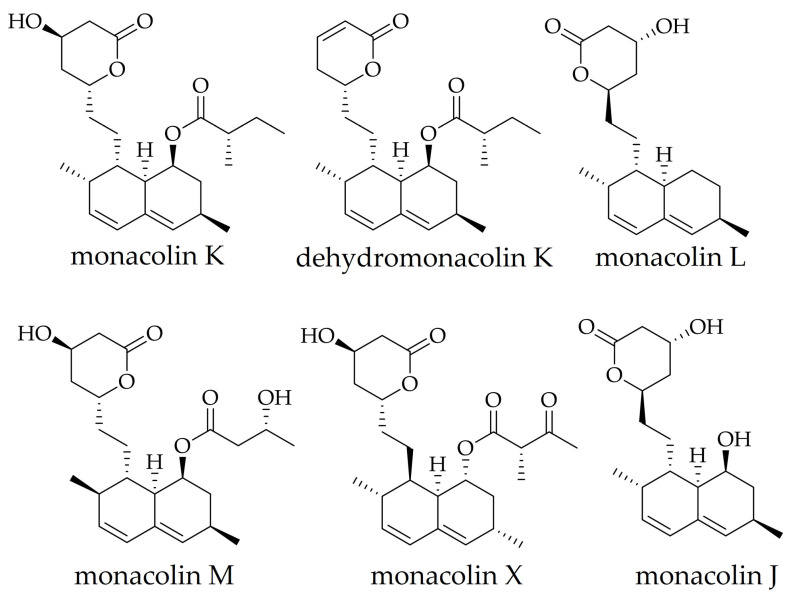
Structures of the main monacolins isolated from *M. purpureus*.

Species of the genus *Monascus* produce a variety of compounds known as monacolins. Monacolins, initially isolated from *M. purpureus*, are monacolin K, J, L, M, X, and dehydromonacolin K. Their structures are shown in Figure 1. Dihydromonacolin L, compactin, and 3α-hydroxy-3,5-dihydromonacolin L are also declared in *M. purpureus* [26]. Monacolins share a common structural scaffold—[8-[2-(4-hydroxy-6-oxo-oxan-2-yl)ethyl]-3,7-dimethyl-1,2,3,7,8,8a-hexahydronaphthalen-1-yl]—and differ mainly in the nature of the substituent at the first position. Among these polyketide-derived metabolites, MK and its dihydro analogue exhibit the highest pharmacological activity [27].

The isolation of monacophenyl, monacolin P and monacolin O from RYR was reported by Liu et al. [28,29]. Subsequently, Zhu et al. identified two novel dehydromonacolins—dehydromonacolin N and dehydromonacolin J, and also defined an additional nine monacolins, including α- and β-dehydrodihydromonacolin K and L, along with the ethyl ester of MK [30]. Thereafter, monacolin R, monacolin S, monacolin Q, α,β-dehydro-monacolin S, α,β-dehydro-monacolin Q, monacolin U, monacolin T, 6a-O-methyl-4,6-dihydromonacolin L, and 6a-O-ethyl-4,6-dihydromonacolin L were found [31,32]. Among all, MK is the most prevalent. During the fermentation process, the content of MK, citrinin (a nephrotoxic metabolite), pigments, and other compounds could be modulated by some modifications applied, such as pH, temperature, nitrogen source, and water supply [33].

**Figure 2:**
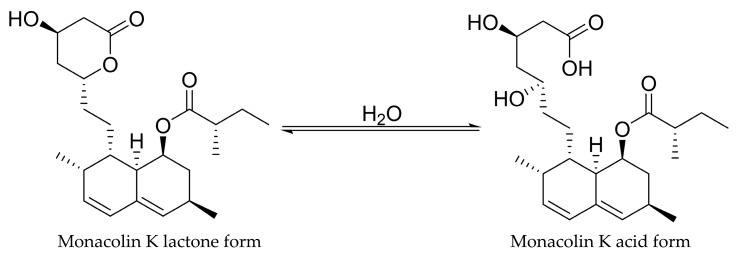
Monakolin K lactone and acid form.

In RYR, MK is present in both lactone (MKL) and hydroxy acid forms (MKA) as shown in Figure 2. It is considered that these two forms exist in a pH-dependent equilibrium: under acidic conditions, the lactone form predominates, while neutral to alkaline conditions favor the hydroxy acid form. The slowest interconversion occurs at approximately pH 4.5. Analyses of RYR samples indicated that MKL generally predominates over the hydroxy acid form in most preparations, highlighting the variability of these compounds depending on processing and environmental conditions [24]. Prior to the drying process, the acid form of MK in RYR is approximately 80%. During drying (temperature 50–60 °C), part of the MKA is converted to the MKL. Over time, the ratio between these two forms remains stable, with the MKL comprising a predominant of 85% [24].

Also, the time of culture growth has an impact on the metabolite content. The levels of MKA, MKLand pigments demonstrated a time-related increase [34]. Selection of strains with minimal citrinin production through mutation methods could also be used, and chemical or physical methods to reduce the concentration of the toxic metabolite and maintain the concentration of MK. Mutagenic strains *M. purpureus* N 301 and N 310 were derived from the parent strain *M. purpureus* NTU 601. These strains exhibited significantly reduced citrinin production while maintaining MK production compared to the parent strain. Specifically, *M. purpureus* N 301 produced 0.01 ppm citrinin and 7.98 ppm MK, and *M. purpureus* N 310 produced 0.01 ppm and 5.54 ppm, respectively [35]. The concentrations of MK and citrinin under optimal conditions in *Monascus* spp. M12-69 mutant strain are 2.52 mg/g and 0.13 ng/g, respectively [36].

Consistent with the broader behavior of fermented products, the MK content of RYR supplements can vary widely depending on the strain and fermentation conditions. While traditionally produced RYR may contain relatively low MK levels, selected or engineered “mutant” strains can generate products with substantially higher concentrations. As a result, some supplements may deliver MK amounts approaching prescription statin doses, with potential consumer risks resembling those associated with statin therapy [10].

Although monacolin K is the principal bioactive compound in red yeast rice, its presence alone is insufficient to confirm product authenticity, as synthetic lovastatin is chemically identical. In contrast, the simultaneous presence of multiple minor monacolins reflects the polyketide biosynthetic activity of *Monascus* species and therefore supports a natural fermentation origin. However, such profiles should be regarded as supportive but not conclusive evidence of authenticity, as they may be altered by purification or blending practices. Consequently, monacolin profiling is best applied as an initial screening step within a broader, multi-level authentication strategy.

### 2.2. Citrinin

**Figure 3:**
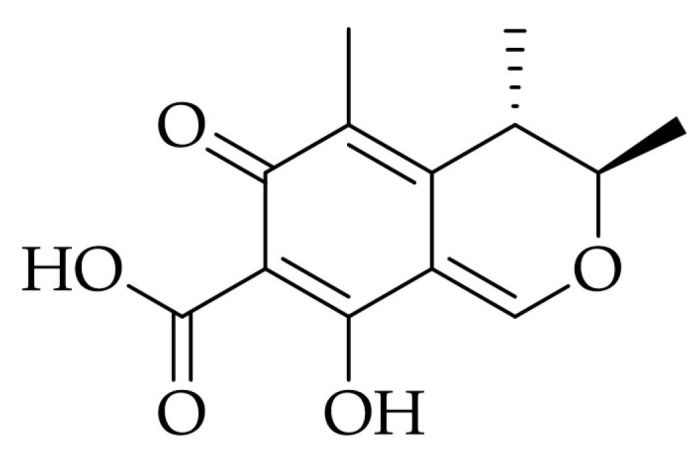
Structure of citrinin.

**Figure 4:**
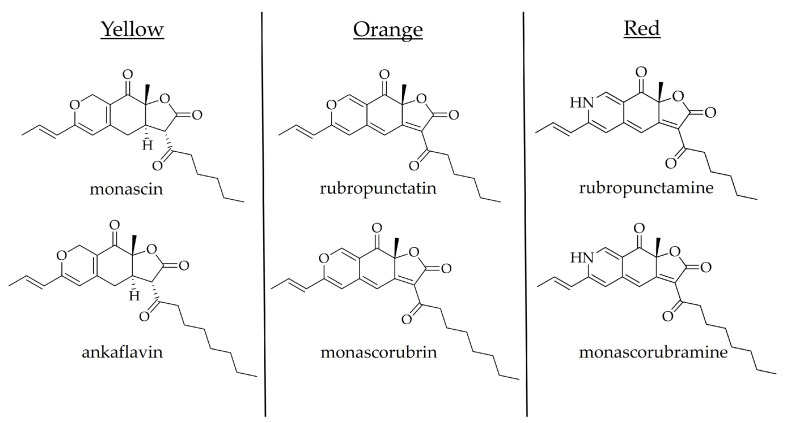
Chemical structures of *Monascus* pigments.

Citrinin (Figure 3), 4,6-dihydro-8-hydroxyl-3,4,5-trimethyl-6-oxo-3H-2-benzopyran-7-carboxylic acid, is a polyketide mycotoxin which was first isolated from *Penicillium citrinum*. It is known to be an incredibly toxic, carcinogenic and mutagenic metabolite [37]. Citrinin is a hepatotoxic metabolite, as it could be a cause of hepatic and extrahepatic carcinogenesis, nephro- and neurotoxic, but also has antimicrobial activity against Gram-negative and Gram-positive microorganisms [26].

Citrinin could also be produced as a secondary metabolite during the fermentation process by *Monascus* spp. [26]. Its amount in solid and submerged cultures is in the range of 100–400 mg/L. In different types of rice, the content can vary from 0.2 to 17.1 ppm [35]. Other organic acids isolated from RYR are linoleic acid, α-linolenic acid, 1-heptadecanecarboxylic acid, 1-pentadecanecarboxylic acid, 2-hydroxyoctadecanoic acid, and 5-(2′-hydroxy-6′-methyl phenyl)-3-methylfuran-2-carboxylic acid, and also two amino acids-(−)-monascumic acid and (+)-monascumic acid [6].

Citrinin has historically served as a key safety and quality marker for red yeast rice products due to its frequent co-production during *Monascus* fermentation and its well-documented nephrotoxicity. However, recent reports of severe adverse events associated with citrinin-free products demonstrate that the absence of citrinin alone cannot be regarded as evidence of product safety or authenticity. The detection of puberulic acid and other blue-mold–derived metabolites in implicated supplements highlights a broader contamination risk that extends beyond traditional *Monascus*-related mycotoxins. Consequently, effective authentication and safety assessment of red yeast rice products should rely on multi-toxin surveillance rather than exclusive dependence on citrinin monitoring.

### 2.3. Pigments

For centuries, rice fermented with *Monascus* has been traditionally employed as a natural food colorant and as a preservative for meat and fish [38]. *Monascus* pigments, which have a color range from yellow to red, have been extensively studied for their potential health-promoting properties and are associated with different biological activities, including antioxidant activity at concentrations of 10 μg/mL or higher, antimicrobial activity against Gram-positive and Gram-negative bacteria, and fungi, and others [39].

The presence of *Monascus* pigments (Figure 3) in the analyzed samples is an important indicator of RYR product authenticity.

These compounds are both polyketides and azaphilones, with bicyclic oxygenated rings and a quaternary center. *Monascus* spp. produce six main pigments—ankaflavin and monascin (yellow), monascorubrin and rubropunctatin (orange), monascorubramine and rubropunctamine (red), and other minor pigments (e.g., xanthomonasins A and B) isolated under different conditions [40]. Pigment profiles could vary significantly depending on *Monascus* spp. and strain, the nitrogen source (amine availability), and the fermentation conditions. pH influences pigment levels, as an acidic pH (2–4) in the culture medium reduces the production of red pigments, resulting in a dominance of orange and yellow pigments [41]. The content of pigments in RYR is nearly 3% [24].

Monascorubrin (Figure 4) is one of the characteristic pigments produced by *Monascus* species during fermentation, commonly found in RYR.

### 2.4. Sterols

Several phytosterols have been isolated, such as stigmasterol, ergosterol, β-sitosterol, β-sitosteryl palmitate, daucosterol, 3β-hydroxylstigmast-5-en-7-one, 6β-hydroxystigmast-4-en-3-one, and others, also contributing to the lipid-lowering effects of red rice [6,42,43]. Phytosterols have been demonstrated to significantly reduce triglycerides, low-density lipoproteins, and total cholesterol levels by approximately 30%. Additionally, an increase in serum high-density lipoprotein cholesterol levels was observed [44].

### 2.5. Decalin Derivatives

Decalin derivatives inhibit human T-cell proliferation by suppressing cell proliferation without exerting cytotoxic effects on these cells [45]. Such decalin derivatives with immunosuppressive activity, isolated from RYR, are monascusic acid A, B, C, D, and E, monascusic lactone A, and heptaketide [31,45,46]. Moreover, the immunosuppression exhibits a dose-dependent effect, with concentrations ranging from 10 µmol/L to 100 µmol/L [45].

### 2.6. Polysaccharides

*Monascus* spp. produce polysaccharides, which have been identified as extracellular and cell wall–associated heteropolymers, formed mainly of glucose, galactose, mannose, rhamnose, and xylose. The monosaccharide composition varies according to the strain and the fermentation process conditions [47,48,49]. Two exopolysaccharide fractions, EPS-1 and EPS-2, were separated in the liquid fermentation of *M. purpureus*. EPS-1 consists primarily of mannose, glucose, and galactose. In contrast, EPS-2 contains additional sugars, including rhamnose, arabinose, and xylose, which indicates significant structural diversity [49]. The extracted alkaline mycelium polysaccharides (MPS) from *M. purpureus* were fractionated into four separate fractions: MPS-1, MPS-2, MPS-3, and MPS-4 [49]. Both EPS-1 and specific MPS fractions (especially MPS-2) showed a significant impact on macrophage proliferation, pinocytosis, and secretion of cytokines such as IL-6, TNF-α, and IL-10, along with increased expression levels of related mRNAs in RAW 264.7 cells, which supports their immunomodulatory potential [49,50]. Furthermore, antioxidant, hypolipidemic, hypoglycemic, and anti-inflammatory activities of other *Monascus* polysaccharide fractions have been demonstrated, related to their specific structural characteristics [48,51,52].

### 2.7. Isoflavones, Lignans, Coumarin, and Terpenoids

The isoflavones daidzein and genistein were isolated from RYR [6]. An isoflavone-enriched fraction has been shown to reduce triglyceride levels by approximately 50%. This fraction, together with RYR extract, increased the reverse transport of cholesterol from the periphery to the liver, increased bile acid (BA) synthase (CYP7A1) levels, i.e., stimulated the conversion of cholesterol to BAs, increased BA secretion, and inhibited BA reabsorption [44]. Furthermore, lignans (lariciresinol, 5,5′-dimethoxycariciresinol), coumarins (scopoletin), terpenoids (α-cadinol, anticopalol, 3-epi-betulinic acid, and others) were identified [6,42,53].

## 3. Market Surveillance and Quality Issues in RYR

### 3.1. Market Surveillance

Market surveillance studies consistently demonstrate substantial variability in the composition and labeling of RYR FSs. Reported MK contents range from negligible amounts to levels approaching or exceeding therapeutic doses. Many products show poor agreement between measured and declared MK content, and in numerous cases, monacolin content is not declared at all. Such discrepancies raise concerns regarding product standardization, consumer transparency, and safety.

Across market surveys, RYR supplements show substantial variability in monacolin content, frequent labeling gaps, and intermittent findings relevant to safety and adulteration. A summary of the main types of adulteration reported in RYR food supplements, the analytical methods used for their detection, and their limitations is presented in Table 1. Collectively, these studies highlight inconsistent quality and reinforce the need for rigorous authentication and quality control strategies.

The data summarized in the table highlight the considerable variability and frequent quality issues observed in RYR FSs across different markets. In addition, cases of adulteration with pharmaceutical statins (e.g., lovastatin or simvastatin) and the presence of undeclared compounds have been identified. Additionally, atypical monacolin patterns or disproportionately high levels of specific monacolin forms have been reported, which may indicate product manipulation, substitution of raw materials, or non-standardized fermentation practices. Together, these findings underscore inconsistent quality, labeling non-compliance, and potential safety concerns associated with RYR supplements, highlighting the need for rigorous authentication, standardized quality control, and routine contaminant monitoring. A variety of analytical techniques are currently employed for the characterization, quality control, and authentication of RYR, each offering specific strengths but also presenting inherent limitations [10,25,54,55,56,57,58,59,60,61,62,63,64].

Collectively, market-surveillance studies demonstrate that variability, mislabeling, and compositional inconsistency are systemic rather than incidental features of the red yeast rice supplement market. The wide dispersion of monacolin K content, frequent absence of label disclosure, and recurrent detection of undeclared statins indicate that quality deviations occur across product types, brands, and regions. Importantly, these findings suggest that non-compliance is not restricted to isolated manufacturers but reflects structural weaknesses in pre-market control and post-market surveillance. From an authentication perspective, this variability reinforces the necessity of analytical verification for each batch rather than reliance on labeling claims or supplier documentation.

### 3.2. Potential Contaminants in RYR FSs

The chemical composition of RYR is strongly influenced by fermentation conditions. Monacolin and pigment production depend on strain selection, substrate type, pH, temperature, moisture, and nutrient sources. *M. purpureus* has traditionally been used due to its pigment-producing capacity, although other *Monascus* species may exhibit higher MK biosynthetic potential. The fermentation process directly affects MK production. Two main modes are distinguished:submerged fermentation (SmF) and solid-state fermentation (SSF), with SSF usually providing significantly higher MK yields. This advantage is attributed to more favorable conditions for fungal growth, metabolic activity, and substrate utilization. Substrate selection also affects MK production, as structural properties of solid matrices (e.g., water absorption, particle size, and viscosity) influence *Monascus* growth and metabolism. In SSF, supplementation with carbohydrate and nitrogen sources (e.g., glucose, glycerol) and metal ions (Mg^2+^, Mn^2+^) considerably increases MK production. In SmF media, optimizing combinations of carbohydrate sources, nitrogen sources, inducers, and precursors (such as linoleic acid and citrates) can also enhance MK levels. Careful control of physicochemical parameters—temperature, water content, pH, and fermentation time—is therefore essential [65]. During SmF with *M. ruber* BCRC 31535, the highest MK content was observed after 120 days of fermentation, which was approximately 5.7 times higher than after 60 days. Also, the expression of genes responsible for MK biosynthesis showed similar dynamics [66]. MK production is primarily determined by strain genetics rather than fermentation conditions alone. *M. ruber* is the principal MK-producing species during fermentation. In contrast, *M. purpureus*, although capable of fermentation, is genetically unable to produce MK if the required biosynthetic gene cluster is absent. Consequently, optimization of fermentation parameters cannot increase MK yield in such strains. Pigment production, however, remains relatively stable and is less sensitive to fermentation conditions. Citrinin production occurs only in strains possessing the relevant biosynthetic potential, allowing toxin control through targeted species and strain selection [67]. SSF provides improved oxygen and nutrient transfer to the mycelium, thereby promoting the synthesis of secondary metabolites such as MK. Under optimized SSF conditions, *M. ruber* produced significantly higher MK levels (14.5 mg/g) than commercial red fermented rice (8 mg/g) [68]. Furthermore, fermentation with NaCl significantly reduced citrinin production, while MK and pigment production increased approximately 1.4-fold compared to the control without NaCl [69]. Moreover, glutinous rice is the most common substrate, although alternative cereals and soybean matrices can also be used. Organic nitrogen sources such as monosodium glutamate, peptone, and yeast extract generally stimulate secondary metabolite production more effectively than inorganic sources, while balanced carbon availability supports fungal growth and sporulation. Optimal MK production typically occurs at moderate temperatures (23–30 °C), near-neutral pH (6–7), and controlled moisture levels. However, conditions that enhance MK synthesis may simultaneously promote citrinin formation, making process control critical for balancing yield and safety [70]. Fermentation substrates and culture conditions play a key role in balancing beneficial and undesirable metabolites. Dioscorea root, for example, can stimulate MK production but may also enhance citrinin formation if not carefully controlled. In SmF with *M. purpureus*, dioscorea-based media increased both MK and citrinin compared to rice media. Response surface methodology studies showed that culture pH, substrate concentration, and ethanol supplementation strongly influence this balance. Moderate pH, reduced substrate concentration, and low ethanol supplementation improved the MK-to-citrinin ratio, demonstrating that toxin risk is both strain- and process-dependent [71]. Quality evaluation should also integrate safety surveillance for fermentation-derived contaminants, especially citrinin. Routine testing frameworks, therefore, combine monacolin profiling with mycotoxin screening, strengthening authentication by addressing both compositional consistency and contaminant risk [63,72]. Moreover, post-fermentation handling and storage also influence RYR quality. MK is susceptible to temperature- and oxygen-dependent degradation. Lower storage temperatures and vacuum packaging improve MK retention, whereas higher temperatures and air exposure promote conversion to dehydromonacolin K and oxidized derivatives. The acid form appears particularly temperature-sensitive. These findings highlight that storage and packaging are critical for maintaining product stability and efficacy [73].

RYR’s cholesterol-lowering effect is mainly due to MK, particularly its MKL, which is chemically identical to lovastatin and therefore associated with a similar side-effect profile. This equivalence has prompted regulatory restrictions on MKL-rich RYR products in countries such as the USA and Canada. MK occurs in two interconvertible forms, MKL and MKA. MKL is pharmacologically inactive and requires in vivo enzymatic hydrolysis to MKA, potentially adding metabolic load to the liver and kidneys, while MKA is the active HMG-CoA reductase inhibitor and is often considered metabolically more favorable. Despite this, most commercial RYR products are dominated by MKL, with EFSA indicating a typical composition of about 85% MKL and 15% MKA, a distribution that may influence both efficacy and safety [74]. However, the predominance of the lactone form should be regarded only as a supportive indicator rather than definitive proof of authenticity. In naturally fermented RYR, the MKL typically does not exceed 70% of the combined lactone and hydroxy-acid forms; higher proportions may raise suspicion of adulteration and warrant isotopic verification [26]. Klingelhöfer et al. reported that fermentation conditions (e.g., pH and nutrient sources) strongly influence pigment and monacolin profiles, and that lactone-to-hydroxy-acid interconversion occurs via lactone-ring opening. In the tested samples, the hydroxy-acid form appeared in most products and the lactone form in nearly all. Citrinin was not detected at the reported limit of quantification. Total monacolin content varied widely, and some products contained high proportions of the hydroxy-acid form, emphasizing the value of monitoring both total monacolins and the lactone:hydroxy-acid ratio [75]. It is also reported that 16 of 32 products had profiles consistent with adulteration, and even non-adulterated products varied considerably. MK and its acid form ranged widely, secondary monacolins were often abundant, citrinin was sometimes detectable, and “total monacolins” frequently overstated true MK and MKA content. Purification and standardization can improve safety and consistency. The purified extract (MonaKoPure-K20) provides over 20% MK and MKA, under 50 ppb citrinin, and minimal secondary monacolins, with total MK reflecting only MK and MKA. These findings highlight the need for stricter quality control [76].

RYR safety depends not only on monacolin levels but also on secondary metabolites from *Monascus* fermentation, especially mycotoxins. Citrinin is one of the main contaminants and a key safety marker; it is nephrotoxic and hepatotoxic and has been widely reported in RYR, with levels varying by strain and fermentation conditions. Because it can co-occur with monacolins, monitoring and regulatory limits (e.g., in the EU) are important. Fermentation parameters (substrate, pH, temperature, ethanol) can help reduce citrinin while preserving bioactives. The European Commission has established a tolerable daily intake of 0.2 μg/kg body weight per day for citrinin, corresponding to a level of no concern for nephrotoxicity [77]. In addition, under Regulation (EU) 2023/915, a maximum concentration of 100 μg/kg has been set for citrinin in RYR FSs to ensure effective protection of public health (Regulation (EU) 2023/915). Given citrinin’s nephrotoxicity and frequent presence in foods, regular RYR consumption can be concerning if contamination occurs. Reliable detection is supported by multiple validated methods, including advanced extraction, clean-up, and analytical techniques (e.g., HPLC, immunoassays, biosensors), enabling better monitoring, risk assessment, and consumer protection [25,64,78,79]. EFSA classifies citrinin as genotoxic, and safety assessment follows a margin-of-exposure approach. For genotoxic compounds, a margin of exposure over 10,000 is considered of low concern. Using a reference body weight of 60 kg, 34 of 37 samples exceeded the level of no concern (0.2 μg/kg b.w./day), with margin-of-exposure values below safety thresholds for nearly all products. Some products labeled “citrinin-free” still posed risk, underscoring the need for stricter regulation and surveillance. Biomonitoring (e.g., urinary citrinin) could refine exposure estimates [25]. Citrinin has been intermittently but repeatedly detected across commercial products, with reported occurrences in 7 from 9 capsule products, 4 from 12 capsule supplements, and 10 from 31 raw materials, although some studies found no detectable levels in finished products [10,59,61,62]. The widest concentration range was reported in a survey of 37 RYR supplements, where citrinin levels ranged from 100 to 25,100 μg/kg, and only one product complied with the 100 μg/kg limit [25]. Market surveys illustrate marked variability across regions and products: citrinin was not detected in 15 Polish supplements, whereas 27 of 77 Taiwanese supplements were positive (0.1–15.2 mg/kg). In China, citrinin was found in 7 of 11 commercial products (maximum 57.28 μg/g) [64,80,81,82]. Contamination with citrinin must be monitored and should accompany authentication and MK quantification. Owing to its conjugated, intrinsically fluorescent structure, HPLC with fluorescence detection remains a common routine approach for citrinin screening and quantification, while LC–MS/MS is increasingly used for confirmation in complex supplement matrices [31].

While prolonged fermentation and poor hygiene have been implicated in toxin formation, recent attention has focused on puberulic acid—a mycotoxin associated with Penicillium “blue mold”—as a possible contributor to RYR-associated kidney injury cases. Puberulic acid is therefore emerging as an additional safety marker alongside citrinin [83]. In early 2024, an incident linked multiple deaths and hospitalizations to RYR supplements. Investigations indicated that toxicity was mainly associated with puberulic acid, a blue mold–derived metabolite, leading to product recalls and halted production. Notably, some implicated products were citrinin-free, highlighting the limitations of relying solely on citrinin as a safety marker. Health authority investigations detected puberulic acid and other blue mold metabolites, and two novel compounds possibly related to lovastatin. Experimental and clinical evidence suggests puberulic acid may contribute to acute and renal toxicity, with kidney dysfunction and Fanconi syndrome reported in some consumers. Overall, these findings indicate that RYR safety assessment should extend beyond citrinin testing [84,85,86,87].

## 4. Analytical Techniques for Authentication and Quality Control

RYR is a chemically complex fermented matrix, and its authentication and quality control require robust, multi-analytical approaches. Advanced techniques such as HPLC, LC–MS/MS, UHPLC–QTOF-MS, NMR, voltammetry, GC–MS, and isotope ratio mass spectrometry consistently reveal substantial product-to-product variability in monacolin profiles, pigment composition, and overall chemical fingerprints.

Fingerprint data frequently reveal additional monacolin analogues and derivatives (e.g., dehydro-/dihydromonacolin K, monacolin L, and hydroxy-acid forms/esters), highlighting the need for robust characterization when comparing products. Where required, orthogonal confirmation (mass spectrometry (MS), NMR, and infrared spectroscopy (IR) in selected cases), together with forced-degradation and accelerated stability studies, can clarify storage-related changes and shelf-life expectations; monitoring multiple monacolins and hydroxy-acid forms provides a more complete picture of chemical variation than MK alone [59,88].

Chromatographic methods coupled with diode-array detection or mass spectrometry and NMR spectroscopy allow rapid, non-destructive compositional profiling with minimal sample preparation and can simultaneously characterize multiple compound classes. GC–MS remains valuable for detecting volatile compounds and certain synthetic adulterants, although derivatization steps may increase analytical complexity. UHPLC–QTOF-MS and HPTLC are often used for chemical fingerprinting and comparative profiling. Despite their strengths, these methods have limitations. High-resolution MS systems require significant investment and expertise. Matrix effects such as ion suppression or enhancement can affect LC–MS/MS quantification in complex natural matrices. Spectroscopic methods may suffer from signal overlap and generally offer lower sensitivity than MS-based techniques. Additionally, structurally similar compounds cannot always be unequivocally distinguished by a single method. Consequently, no individual analytical technique is sufficient to fully address RYR complexity, and complementary multi-technique strategies are recommended [89,90,91]. These approaches enable the detection of monacolins, mycotoxins, pigments, and potential adulterants with good precision and sensitivity. LC-based profiling is particularly valuable because it captures MK together with other bioactive and undesirable constituents, typically present as mixtures of related compounds in both lactone and hydroxy-acid forms. This multi-component behavior supports a profile-based approach for identity verification and dose consistency rather than reliance on single-marker testing [92].

A major analytical challenge is adulteration with pharmaceutical lovastatin, which is chemically identical to MK and therefore indistinguishable by routine LC-based quantification. Product-to-product variability in total monacolins and lactone-to-hydroxy-acid distribution may further complicate interpretation and contribute to variable bioavailability.

To verify origin, stable isotope ratio analysis (δ^13^C) by EA-IRMS after chromatographic isolation has been proposed as a confirmatory tool. δ^13^C values reflect biosynthetic carbon sources: RYR-derived MK typically exhibits C3 plant signatures, whereas industrial lovastatin often shows a C4-shifted profile associated with corn- or cane-derived substrates. Because isotopic signatures directly reflect biosynthetic pathways, δ^13^C-IRMS is currently considered the most reliable approach for distinguishing natural MK from synthetic lovastatin and identifying fraudulent additions. Rapid screening methods such as flow-injection MS/MS (FI–MS/MS) using multiple MRM transitions and ion-ratio checks can serve as efficient triage tools, with confirmatory LC–MS/MS applied for definitive assessment Rapid triage methods, such as FI–MS/MS (with multiple MRM transitions and ion-ratio checks) can screen supplements efficiently, with confirmatory LC–MS/MS used for definitive assessment [27,93,94,95]. Studies demonstrate that isotopic ratios can detect fraudulent addition of synthetic, petroleum-derived lovastatin to RYR products [27].

## 5. Authentication Strategy

Based on the analytical challenges and sources of variability discussed above, a structured authentication strategy is necessary to ensure the identity, quality, and safety of RYR products. Because no single analytical method is sufficient to address all aspects of RYR complexity, a stepwise, multi-technique approach is recommended.

The initial step in the authentication of RYR products should involve quantifying the MK content to verify whether it matches the levels indicated on the product label. Although the presence of other monacolins like J, M, L, N, X, and dehydro-/dihydromonacolin K would confirm that the origin of MK is natural. The subsequent step should focus on the identification of the monascorubrin pigment as a characteristic marker of RYR.

Gas chromatography–mass spectrometry (GC-MS) analyses should be obligatory performed in full scan mode for confirmation of the presence of undeclared compounds such as simvastatin, rosuvastatin and atorvastatin. RYR is generally well tolerated at MK doses up to 10 mg/day, though mild effects such as myalgia have been reported. EFSA approved a health claim in 2011 for RYR providing 10 mg MK for cholesterol control, but in 2018 noted that MKL is identical to lovastatin and can cause similar side effects. EFSA therefore reduced the recommended daily intake from 10 mg to less than 3 mg. Indeed, if samples are contaminated with other statins, the total statin intake may increase, potentially leading to more pronounced adverse effects [96].

An additional analysis that could be performed for confirmation of the authenticity is ^1^H-NMR. Although lovastatin and MK cannot be distinguished with this technique, ^1^H-NMR allows the comprehensive profiling of compounds in RYR products through a single analysis of the crude extract. This technique can simultaneously detect all monacolins, pigments such as monascin and structurally related pigments, fatty acids (saturated and unsaturated), polyols (e.g., glycerol, sorbitol), glucose, and additional formulation components often included in supplements, such as piperine, carnitine, and vitamins. A key limitation of this approach is that the observed resonances typically correspond to classes of compounds rather than individual molecules. For instance, this approach distinguishes monacolins containing a hexahydronaphthalene ring but cannot differentiate MK specifically, and it identifies monascin along with other pigments sharing the same structural skeleton rather than monascin alone [58].

RYR products present significant quality and safety challenges due to their complex and variable nature. The fermentation process, which depends on the Monascus strain and cultivation conditions, leads to substantial natural variability in the chemical composition of RYR, resulting in inconsistent levels of bioactive and secondary metabolites. In particular, the presence of pharmacologically active MK—chemically identical to the prescription drug lovastatin—raises safety concerns, as its concentration may approach therapeutic doses without appropriate medical supervision. In addition, RYR is susceptible to contamination with the nephrotoxic mycotoxin citrinin, which may be co-produced during fermentation. Finally, cases of adulteration with synthetic statins or other undeclared substances have been reported, further complicating quality control and increasing the potential risk to consumers.

**Figure 5:**
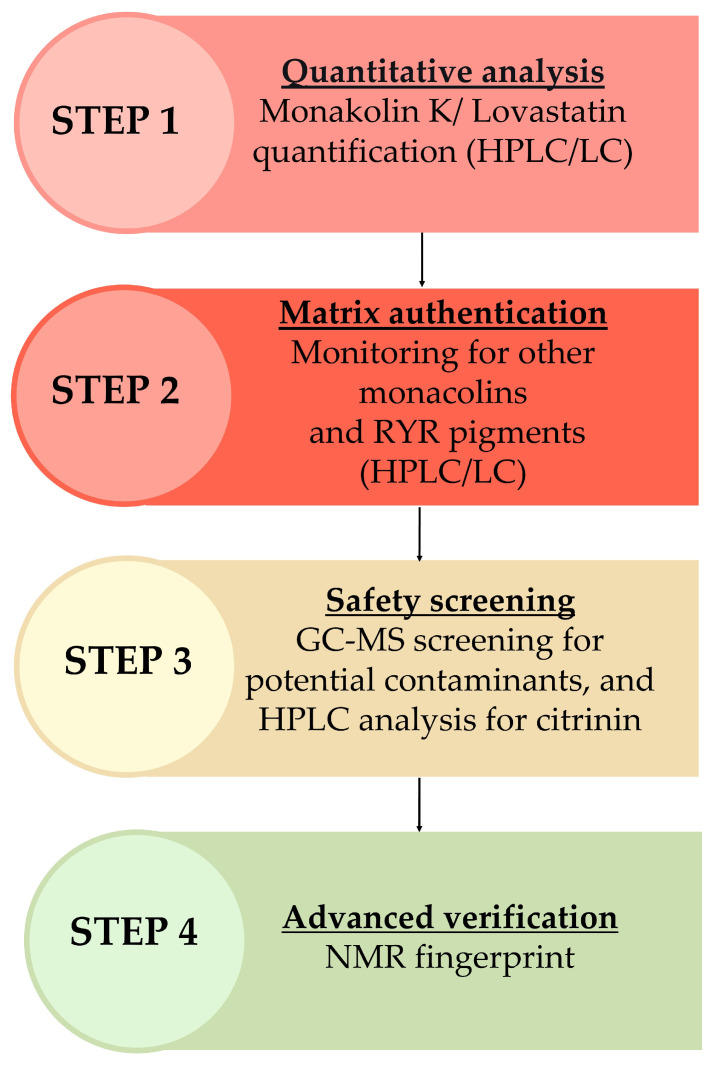
Analytical workflow for authentication of RYR products.

The authentication of RYR products is a complex, time-consuming, and often costly process. Nevertheless, it is essential to ensure consumer safety. Despite the relatively liberal regulatory framework governing FS in the EU, manufacturers of RYR-based products bear a significant responsibility to guarantee product quality, consistency, and safety. Given the natural variability of RYR preparations and the potential presence of undeclared or harmful constituents, a rigorous and well-defined analytical strategy is required. We therefore propose a comprehensive, stepwise analytical workflow (Figure 5) designed to support the authentication, quality control, and safety assessment of RYR FSs. This workflow integrates complementary analytical techniques to enable the reliable characterization of active components, pigments, potential contaminants, and overall compositional fingerprints. To ensure transparency, reproducibility, and batch-to-batch consistency, manufacturers/importers of RYR FSs should establish and provide validated analytical protocols and corresponding results for each production batch.

## 6. Conclusions

The authentication of RYR products involves a multi-analytical approach strategy. The combination of different analytical techniques is crucial for the quality assessment of these products. ^1^H-NMR allows a comprehensive overview of all compounds in RYR FSs through a single analysis of the crude extract. The primary limitation of this method is that the detected resonances generally correspond to classes of compounds rather than individual molecules. HPLC techniques allow accurate characterization of target compounds. Nonetheless, like other separation-based methods, it requires considerable time and preparation, and the chosen operating conditions are generally tailored to specific compound types, for example, monacolins and pigments, limiting the analysis to these classes. GC-MS complements HPLC and NMR by detecting volatile, semi-volatile, and thermally stable compounds that other methods may miss. It provides structural identification, quantification, and detection of minor metabolites or contaminants, enhancing both authenticity verification and quality control. Together with HPLC (targeting monacolins and pigments) and NMR (providing a global metabolite fingerprint), GC-MS ensures a comprehensive chemical characterization of RYR products. The development of standardized analytical workflows for the authentication of RYR products offers a clear opportunity to support future regulatory guidelines and harmonized quality standards. By integrating complementary targeted and non-targeted analytical techniques, such approaches could serve as a reference framework for manufacturers, quality control laboratories, and regulatory authorities. Establishing minimum testing requirements, including batch-specific documentation and validated analytical methods, would improve transparency, reproducibility, and consumer protection. Ultimately, the adoption of standardized strategies may facilitate more consistent regulatory oversight and contribute to the safer and more reliable use of RYR-based FSs.

## Figures and Tables

**Table 1:** Adulterations, quality-control findings, and analytical methods applied to RYR FSs.

Analytes Target	Supplement Type	Analytical Technique	Analytes Found	Ref.
Monacolin K	28 FSs: tablets and capsules.	UHPLC-DAD-QTOF-MS	Among the brands analyzed, MK content ranged from 0.09 to 5.48 mg per 1200 mg of RYR. Ten of the products contained approximately 2 mg MK per 1200 mg RYR. None of the brands disclosed MK content on their labels, and only two advised consumers not to use the supplement concurrently with prescription statins.	[10]
Monacolin K	FSs: tablets (2), gel capsules (1), capsules (2), liquid capsules (1)	Square-wave voltammetric method	Discrepancy between the determined and the labeled content of MK (1.9–153.5%). 3 products contain higher levels of MK than declared, whereas 1 product contains only 1.9% of its stated MK content. 1 of the tested samples does not contain the labeled amount of MK content.	[54]
MKA and MKL	Capsules	High-performance liquid chromatography (HPLC)	The concentration of MKA in the sample was 0.0027 mg/mL, and that of MKL was 0.0035 mg/mL. The concentration of MK in 600 mg red yeast capsule is 2.48 mg/capsule, or 103.3% of the labeled amount.	[55]
MK, MKA, monacolin J acid, dihydromonacolin K, dehydromonacolin K, and α,β-dehydrodihydromonacolin K	10 FSs: tablets (8), capsules (2).	High-performance liquid chromatography (HPLC-MS)	MK content ranged from 1.51 to 9.88 mg per serving. Monacolin J acid, dihydromonacolin K, and dehydromonacolin K predominated in products with the highest MK levels, whereas MK-A was more abundant in products with lower MK content.	[56]
36 monacolins and 8 pigments	33 RYR botanicals, including pure RYR and multi-ingredient formulations with standardized amounts of MK: capsules (17), tablets (13) and soft capsules (3)	UHPLC-MS-QTOF	Minor monacolins average 37% of the total monacolin-pigment content. Dehydromonacolins and dihydromonacolins range from 7–43%, with 26 products containing a relative percentage of over 20%. Pigments range from traces to 65%, with 12 products exceeding 20%.	[57]
12 monacolins and 11 azaphilones	31 RYR DS: tablets (11) and capsules (20)	Proton Nuclear Magnetic Resonance (^1^H-NMR) and UHPLC-DAD-MS	Limited data on monacolin content was provided on the labels of 13 analyzed food supplements. Among the remaining products, only nine contained the declared amount of monacolins.	[58]
Total Monacolins, monacolin L and citrinin	9 capsules	HPLC	Total Monacolins K 0.15 to 3.37 mg per capsule. Citrinin—detected in 7 of 9 samples tested.	[59]
MK, monacolin A and citrinin	10 FSs: film-coated tablets (2), tablets (4), capsules (4)	Reversed-phase high-performance liquid chromatography with diode array detection (RP-HPLC-DAD)	MKA varied and was not detected in several products, where MK was present only as MKL. Among supplements declaring <3 mg MK, measured content ranged from 89.86–105.89% of label claims. One product recommended fractional dosing despite containing 8.83 mg MK per tablet.	[60]
Total monacolins (TM), MK, MKA, and citrinin	12 FSs (capsules)	HPLC-MS/MS	TM: 0.31–11.15 mg/capsule; MK: 0.10–10.09 mg/capsule; MKA: 0.00–2.30 mg/capsule; citrinin detected in 4 of 12 samples tested. The sample with the lowest MK content showed the highest citrinin contamination.	[61]
MK, monacolin K acid, monacolin J, compactin, dehydromonacolin K, citrinin and sudan red G	3 authentic RYR Samples, 31 commercial RYR raw materials, 14 FSs (capsules/tablets)	Ultra-high performance liquid chromatography–diode array detection–quadrupole time-of-flight mass spectrometry (UHPLC–DAD–QToF-MS) with chemometric analysis	Three authenticated RYR samples contained MK at 1.9–2.3 mg/g (*w*/*w*) dry weight and monacolin MKA at 1.3–1.6 mg/g (*w*/*w*) dry weight. Among 31 commercial raw materials, MK was not detected in 10 samples, while it was present in 21 samples at concentrations of 0.7–24.3 mg/g (*w*/*w*) dry weight; MKA in these positive samples ranged from 1.3 to 7.6 mg/g (*w*/*w*) dry weight. Citrinin was detected in 10 of the 31 raw materials. In 14 FSs, MK ranged from 0.05 to 4.37 mg/g (*w*/*w*); citrinin was not detected. Other monacolins were present only at trace levels (up to ~0.02%). Sudan Red G was not detected in any sample.	[62]
Total monacolin K (MK and MKA), and citrinin	35 FSs: tablets (11), capsules with powdered content (22), capsules with liquid content (1), soft gels (1).	LC-MS/MS	3 FSs contained more than 3 mg of monacolins; 3 samples were mixed with lovastatin. All samples met the citrinin content requirements.	[63]
MK and citrinin	37 RYR FSs, including pure RYR and multi-ingredient formulations: tablets (15), capsules (18), and soft capsules (4)	UHPLC-MS/MS	The amount of citrinin in the products ranged from 100–25,100 μg/kg, with only 1 product meeting the limit of 100 μg/kg. Citrinin was found in 4 products labeled as citrinin-free. In 24 products, a higher amount of MK was observed—10–266%, while only 3 products had negligible MK content. The daily dose in 19 products exceeded 10 mg. In 30 samples, small amounts of simvastatin (0.1–7.5 μg/daily dose) were found.	[25]
Citrinin	15 FSs: tablets (8), capsules with powdered content (5), capsules with oily content (1), and sachets (1)	HPLC with fluorescence detection	None of the tested samples contain citrinin.	[64]

## Data Availability

Data is contained within the article.

## Abbreviations

The following abbreviations are used in this manuscript:

Ba	Bile acid
CVD	Cardiovascular disease
EFSA	European Food Safety Authority
EU	European Union
FSs	Food supplements
GC-MS	Gas chromatography–mass spectrometry
HPLC	High-performance liquid chromatography
HPLC/MS	High-performance liquid chromatography coupled with mass spectrometry
IR	Infrared spectroscopy
LC–MS/MS	Liquid chromatography tandem mass spectrometry
MK	Monacolin K
MKA	Monacolin K hydroxy acid form
MKL	Monacolin K lactone form
MPS	Mycelium polysaccharides
MS	Mass spectrometry
NMR	Nuclear magnetic resonance
RYR	Red yeast rice
SmF	Distinguished-submerged fermentation
SSF	Solid-state fermentation
UHPLC–DAD–QToF-MS	Ultrahigh-performance liquid chromatography diode array detector hyphenated to a quadrupole–time-of-flight mass spectrometer
UHPLC–QToF-MS	Using ultra-high performance liquid chromatography–quadrupole time-of-flight mass spectrometry
